# Typical Shape Differences in the Subtalar Joint Bones Between Subjects with Chronic Ankle Instability and Controls

**DOI:** 10.1002/jor.24336

**Published:** 2019-05-26

**Authors:** Nazlı Tümer, Gwendolyn Vuurberg, Leendert Blankevoort, Gino M M. J. Kerkhoffs, Gabrielle J. M. Tuijthof, Amir A. Zadpoor

**Affiliations:** ^1^ Department of Biomechanical Engineering Delft University of Technology (TU Delft) Delft The Netherlands; ^2^ Department of Orthopaedic Surgery, Amsterdam UMC, University of Amsterdam Amsterdam Movement Sciences Amsterdam The Netherlands; ^3^ Academic Center for Evidence‐based Sports Medicine (ACES) Amsterdam The Netherlands; ^4^ Amsterdam Collaboration on Health & Safety in Sports (ACHSS) AMC/VUmc IOC Research Center Amsterdam The Netherlands; ^5^ Research Centre Smart Devices Zuyd University of Applied Sciences Heerlen The Netherlands

**Keywords:** ankle sprain, chronic ankle instability, subtalar joint, statistical shape models

## Abstract

Bone shapes, particularly those defining the subtalar joint (STJ), have not received much attention yet as a risk factor for developing chronic ankle instability (CAI) after sustaining a lateral ankle sprain (LAS). This study aimed to compare three‐dimensional (3D) shape variations in the STJ bones within individuals with CAI and healthy controls. 3D statistical shape models (SSMs) of the STJ bones were built to describe the bone shape variations observed within a population consisting of 26 individuals with unilateral CAI and 26 healthy controls. Using the SSMs and analysis of covariance test, age‐ and gender‐adjusted shape variations in the bones were compared within individuals with CAI and healthy controls. The mean age of the CAI patients (14 males and 12 females) and healthy controls (12 males and 14 females) was 29 (standard deviation [SD] = 11) and 36 years (SD = 11), respectively. Tali and calcanei did not significantly vary between ipsilateral CAI and their contralateral ankle. Two shape modes, one for the talus (*p* = 0.015, variations in the curvature of the talar lateral process and the inclination angle of the talar neck relative to the body) and one for the calcaneus (*p* = 0.003, variations in the medial and lateral tuberosities, and the contour of the anterior articular surface), described significant shape differences between the CAI patients and healthy controls. The CAI patients generally had flatter talar joint surfaces and a flattened calcaneal ground‐contact surface. These findings suggest that specific bone shapes may increase the risk of developing CAI after sustaining a LAS. © 2019 The Authors. *Journal of Orthopaedic Research^®^* Published by Wiley Periodicals, Inc. on behalf of Orthopaedic Research Society. J Orthop Res 37:1892–1902, 2019

Lateral ankle sprain (LAS) comprises approximately 80% of all ankle sprains,^1^, ^2^ leading to an average of two million cases in the United States^3^ annually and an estimated 5% of emergency room visits in the United Kingdom.^4^ The true incidence of LAS is even higher as approximately half of the people sustaining a LAS do not seek professional medical help.^4^, ^5^, ^6^

Most patients experiencing a LAS can be successfully treated and regain functional ankle stability with conservative treatment.^7^, ^8^ Despite recommended conservative treatments (e.g., functional support and exercise therapy),^9^ up to 40% of the patients continue to suffer from residual complaints^10^, ^11^, ^12^, ^13^, ^14^ and may progress to chronic ankle instability (CAI).^15^ CAI is defined as the instability of the ankle with the feeling of giving‐way, episodes of recurrent ankle sprains, with or without the presence of joint laxity.^2^, ^16^

Limited evidence is available suggesting that surgical treatment is more effective in decreasing the frequency of recurrent ankle sprains.^17^ Despite its potential success, surgical treatment is generally reserved for athletes to enable them quickly return to play and is not considered to be the preferred treatment option due to the increased costs and the risk of complications^18^ without knowing whether the LAS will progress to CAI. Considering the clinical evidence that CAI may lead to an early onset of post‐traumatic osteoarthritis,^13^, ^19^ it is important to identify patients at risk of developing CAI who might benefit from a surgical treatment rather than a conservative treatment.

A great deal of effort has been put into identifying the factors associated with the development of CAI (e.g., ligament laxity, muscle weakness, postural control deficits,^2^, ^20^, ^21^ joint congruency,^7^, ^22^, ^23^ fibular position,^5^, ^24^, ^25^ cavus foot deformity,^8^, ^24^, ^26^ and varus ankle or hindfoot^27^). However, the studies of bone shape as a factor^7^, ^22^, ^23^ are limited to simple measurements on two‐dimensional (2D) images that cannot fully reflect the three‐dimensional (3D) nature of bone shape.

As the morphology of articulating bones contributes to the stability of the joints and determines their kinematics,^28^, ^29^, ^30^ morphological variations are expected to change the mechanical environment of the joints and modify the risk of CAI. The bones of the subtalar joint (STJ) including the talus and calcaneus to which the most frequently damaged ligaments in LAS (i.e., anterior talofibular ligament and calcaneofibular ligament) connect, are among the articulating bones that can show morphological variations and may contribute to the risk of CAI. In up to 58% of the cases, instability does not solely appear in the talocrural joint (TCJ), but is also present in the STJ of CAI patients.^2^

Despite the important role of the STJ in the hindfoot and its possible contributions to CAI,^8^ little attention has been paid to the factors that may alter the mechanical environment of the STJ. Considering the scarcity of the studies on shape variations in the STJ bones and their relations with CAI, the main aim of the current study was to systematically characterize the 3D shape variations of the STJ bones (i.e., the calcaneus and talus) and quantitatively compare them in threefold (i.e., CAI vs. CAI contralateral controls, CAI vs. healthy controls, and CAI contralateral controls vs. healthy controls). For this purpose, 3D statistical shape models (SSM)^31^, ^32^ and a number of statistical tools were used.

## MATERIALS AND METHODS

### General Scheme

**Figure 1:**
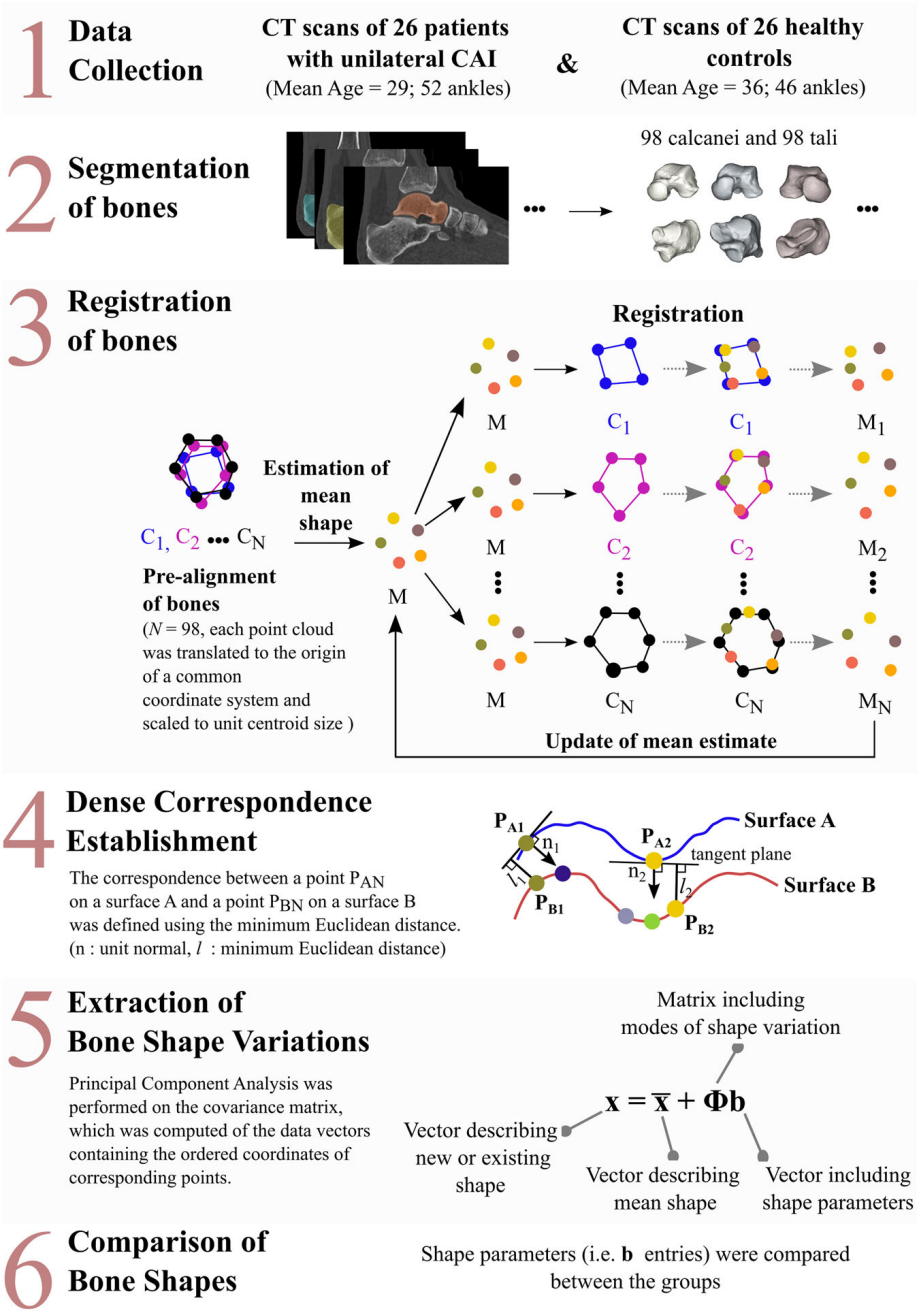
A general scheme to extract the shape variations of the talus and calcaneus and to compare them in three different ways, namely CAI versus CAI contralateral controls, CAI versus healthy controls, and CAI contralateral controls versus healthy controls. CAI, chronic ankle instability, CT, computed tomography. [Color figure can be viewed at wileyonlinelibrary.com].

The main steps involved in the generation of two 3D SSMs (one for the talus and one for the calcaneus) are presented in Figure 1. This workflow is similar to what we have described elsewhere^32^ and includes the following steps: data collection, segmentation and registration of the bones, dense correspondence establishment, extraction of the bone shape variations, and statistical comparison of the shape variations between different groups.

### Data Collection

The computed tomography (CT) scans of a number of patients with confirmed CAI and healthy controls were retrieved from the database of Amsterdam UMC (location AMC, The Netherlands). All CAI patients visited Amsterdam UMC for persistent complaints of ankle instability in combination with recurrent lateral sprains, with or without pain and had undergone non‐surgical treatments at least for 6 months without success. Healthy controls (*n* = 40) were the individuals who had voluntarily undergone CT scanning either as a part of a study investigating the in‐vivo measured range of motion of the STJ^33^ (20 healthy controls) or as a part of a study on the 3D orientation of the posterior facet of the STJ^34^ (20 healthy controls). None of the volunteers had concomitant ankle injury or other joint pathology, recorded complaints, or surgery of the lower extremities. The CT scans were discarded from the study, if the patients were 16 years of age or younger at the time of scanning or if the entire volume of the calcanei was not visible. Thereafter, the CT scans of 14 healthy controls were randomly excluded to have equally sized groups of CAI patients and healthy controls. The final data set included 52 CT scans from 52 subjects (26 patients with unilateral CAI and 26 healthy controls). Six CT scans were unilateral (i.e., 6 healthy controls with left ankles) while the others were bilateral (i.e., 26 patients with unilateral CAI and 20 healthy controls with both left and right ankles). As all CT scans were retrospectively acquired from the CAI patients, the medical ethical committee provided a waiver. Regarding the healthy controls, the participating volunteers had signed informed consents prior to image acquisition and participation in the previously performed studies.^33^, ^34^ Both studies were also approved by the medical ethics committee of Amsterdam UMC.

CT scans were acquired either using a Philips Brilliance 64 or a Phillips MX‐8000 multidetector CT scanner (Philips Medical Systems, Best, The Netherlands). The acquisition parameters (e.g., effective dose, tube voltage) and the tomographic reconstructions showed certain levels of variability within the data set. In particular, the voxel sizes varied between 0.3 × 0.3 × 0.3 mm and 0.7 × 0.5 × 0.5 mm.

### Segmentation and Registration of Bones

All calcanei (*n* = 98) and tali (*n* = 98) were segmented following the same segmentation protocol as used in our previous study.^32^ Some of the steps (i.e., thresholding, labeling bones, filling holes inside segmentation masks, smoothing) were automated using the scripting module of Mimics (version Research 20.0 Alpha; Materialise, Leuven, Belgium). Each segmentation was visually checked to ensure the proper definition of the bony shape contours. Manual corrections were made when deemed necessary. Using the same software, triangulated bone surfaces were extracted from the segmentation results. All instances of the right side tali (or calcanei) were mirrored in the sagittal plane.

The differences between the bone instances due to their relative translations, different orientations, and different scaling factors were minimized by aligning all the tali (or calcanei) using an unbiased registration algorithm.^31^, ^32^ In the alignment procedure (Fig. 1, step 3), each bone sample was first translated to the origin of a common coordinate system and was then scaled to the unit centroid size.^35^ Then, an evolving mean shape was fit to each bone sample described by a point cloud (i.e., a set of points sampled across all the surfaces of the bone sample). The registration parameters that needed to be input by the user (i.e., the scaling parameter for the mixture of Gaussians, *σ*, the number of points in the mean cloud, *n*_m_, and the trade‐off parameter, *λ*) were retrieved from our previously published studies for the tali (*σ* = 3 mm, *n*_m_ = 2,000, *λ* = 10^−6^)^32^ and calcanei (*σ* = 3 mm, *n*_m_ = 2,000, *λ* = 5 × 10^−4^).^35^

### Extraction of Bone Shape Variations

Following the registration process, a dense correspondence (Fig. 1, step 4) across all tali (*n* = 8,667) and calcanei (*n* = 13,220) was established using the coordinates of all points and surface normals as described in a study performed by van de Giessen et al.^36^ (Supplementary Material). We then computed the covariance matrix of the data vectors containing the ordered coordinates (*x*, *y*, *z*) of the corresponding points established across all tali (or calcanei) of the CAI patients as well as those of the healthy controls. Then, a principal component analysis was performed on the covariance matrix. As a result, the modes of shape variation (i.e., eigenvectors) with a descending order of variance (i.e., eigenvalues) were obtained. The modes of shape variation describe the directions of shape changes, while the variance describes how much variation is present in the corresponding direction.

A new or existing talar (or calcaneal) shape (**x**) can be represented, using the mean shape of the talus (or the calcaneus) ($\overset{®}{x}$) and a weighted sum of the modes of shape variation for the talus (or for the calcaneus) (**Φ**), as^37^:

(1) $$x\;=\overset{®}{x}+\sum_{s=1}^{c}b_{s}\Phi_{s}$$

where the *b* values (i.e., shape parameters) describe the contributions of the first *c* modes of shape variation to the mean bone shape. In other words, a shape parameter (e.g., *b*_1_) for a given mode of shape variation (e.g., Ф_1_) describes how far a shape **x** is away from the mean shape $\overset{®}{x}$ in the specified direction (e.g., Ф_1_) (Supplementary Material).

### Comparison of Bone Shapes Between the Groups

While the mean talus (or calcaneus) shape, $\overset{®}{x}$, and the modes of variation, **Ф**, are identical for each talus (or calcaneus) regardless of its group (patients with CAI vs. healthy controls), the shape parameters (i.e., *b* values) are different.^32^ Therefore, for each bone type, the shape parameters (i.e., *b* values) for the first *c* modes of shape variation were compared between the three groups using an analysis of variance test. For pairwise comparisons of the groups (i.e., CAI vs. CAI contralateral controls, CAI vs. healthy controls, and CAI contralateral controls vs. healthy controls), the Bonferroni post‐hoc analysis was performed. Only the shape modes that represented more than 5% of the total shape variation were included.

To evaluate whether the observed variations in the bone shape were affected by age and gender, an analysis of covariance (ANCOVA) test was performed considering the age and gender as covariate factors. To assess whether the shape parameters (i.e., *b* values) across all tali and calcanei for each mode of shape variation conform to a normal distribution, a Kolmogorov–Smirnoff test was applied prior to our statistical analyses. All statistical analyses were carried out using SPSS (version 22; Chicago, IL).

## RESULTS

### Cohort Characteristics

The CAI patients were almost equally distributed in terms of their gender (14 males and 12 females). The mean age of the patients was 29 years (standard deviation [SD] = 11). There were 12 males and 14 females in the healthy control group with a mean age of 36 years (SD = 11). In 62% of the patients, the right ankle was affected by CAI (*n* = 16).

### General Bone Dominant Shape Variations

The number of the modes of shape variation retained for statistical analyses, *c*, was 6 for the talus and 5 for the calcaneus. The analyzed modes (i.e., *c* = 6 for the talus and *c* = 5 for the calcaneus), respectively described 49% and 45% of the total shape variance in the talus and calcaneus (Supplementary Material). For both bone types, the distributions of the shape parameters for the first *c* modes did not significantly differ from a Gaussian distribution (*p* > 0.05).

### Description of Bone Shape Variations

**Figure 2:**
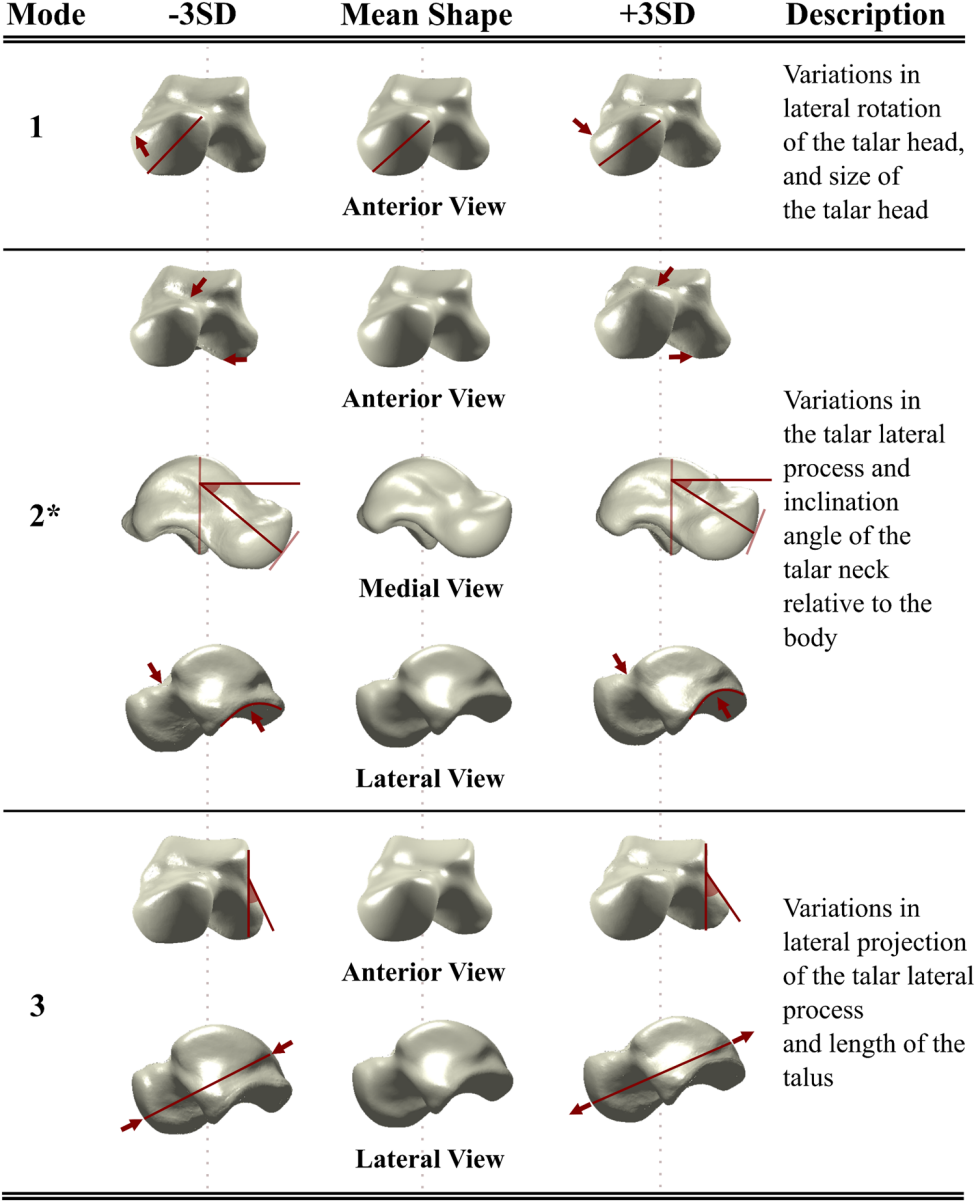
The shape variations of the talus described by its first three shape modes. The red lines and arrows highlight the changes in the shape. The shape mode that is marked with a star was significantly different between the CAI patients and healthy controls (i.e., ipsilateral CAI vs. healthy controls, and CAI contralateral controls vs. healthy controls). CAI, chronic ankle instability; SD, standard deviation. [Color figure can be viewed at wileyonlinelibrary.com].

**Figure 3:**
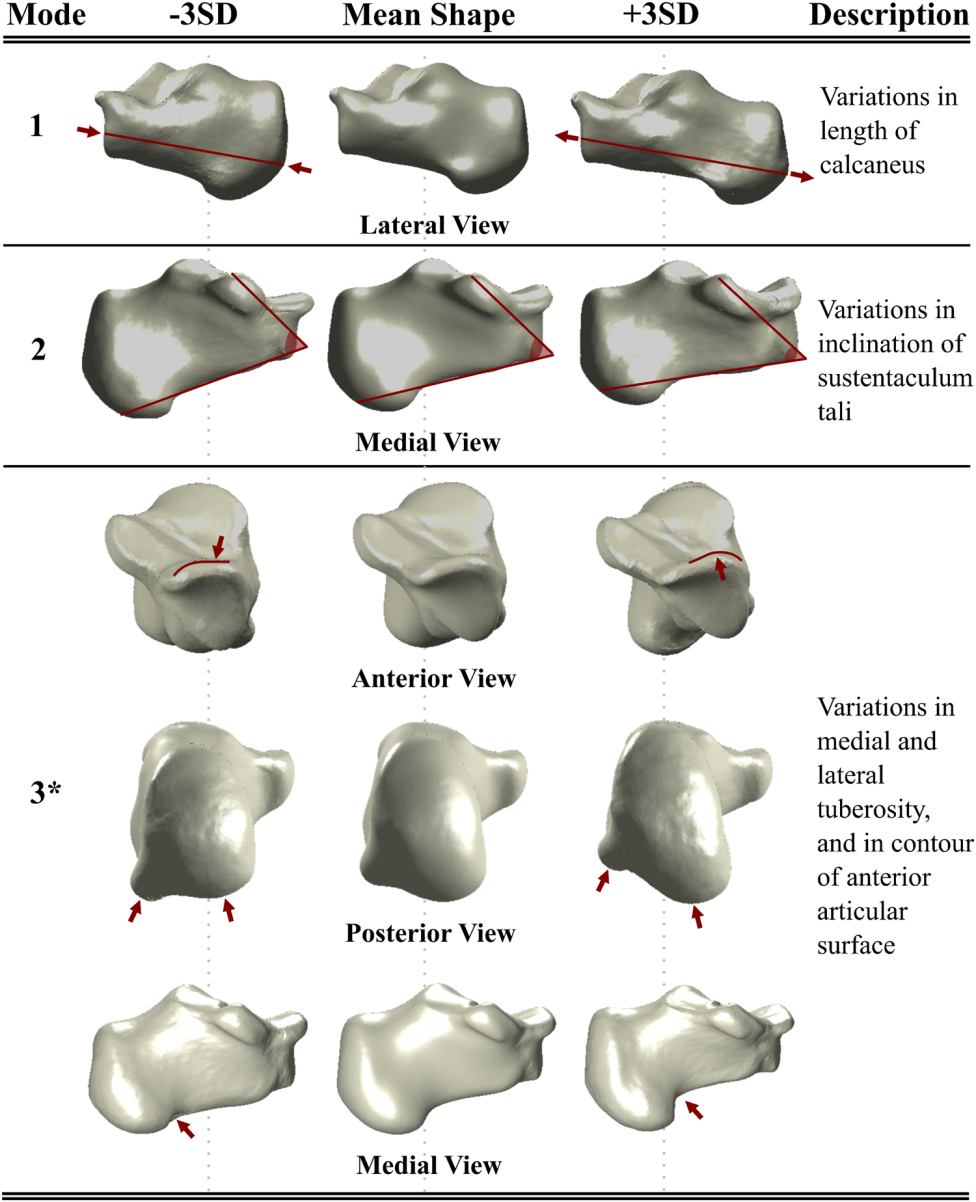
The shape variations of the calcaneus described by its first three shape modes. The red lines and arrows highlight the changes in the shape. The shape mode that is marked with a star was significantly different between the CAI patients and healthy controls (i.e., ipsilateral CAI vs. healthy controls, and CAI contralateral controls vs. healthy controls). CAI, chronic ankle instability; SD, standard deviation. [Color figure can be viewed at wileyonlinelibrary.com].

The descriptions of the bone shape variations are only provided for the first three shape modes of the talus (Fig. 2) and calcaneus (Fig. 3). That is because the shape Modes 4–6 for the talus and shape Modes 4–5 for the calcaneus explained relatively small amounts of shape variations distributed over the bone surfaces and none of them were found to be significantly different in any pairwise comparisons of the three groups.

The first three modes of shape variation of the talus (Fig. 2) and calcaneus (Fig. 3) described 29% and 31% of the total variance in the talus and calcaneus, respectively (Supplementary Material). These modes explained the following changes:

- Talus Mode 1: the lateral rotation and the size of the talar head.
- Talus Mode 2: the curvature of the talar lateral process and the inclination angle of the talar neck relative to the body.^38^
- Talus Mode 3: the lateral projection of the talar process and the length of the talus.
- Calcaneus Mode 1: the length of the calcaneus.
- Calcaneus Mode 2: the inclination of the sustentaculum tali.
- Calcaneus Mode 3: the medial and lateral tuberosity and the contour of the anterior articular surface.

### Comparison of Bone Shape Variations Between Three Groups

The shapes of the talus and calcaneus did not significantly (*p* > 0.05) differ between the ipsilateral and contralateral sides of the subjects who had a unilateral CAI (i.e., CAI vs. CAI contralateral controls).

The shape variations of the talus described by Mode 2 (Fig. 2) were significantly different between the CAI group and healthy controls (*p* = 0.015) as well as between the CAI contralateral controls and healthy controls (*p* = 0.035).

**Figure 4:**
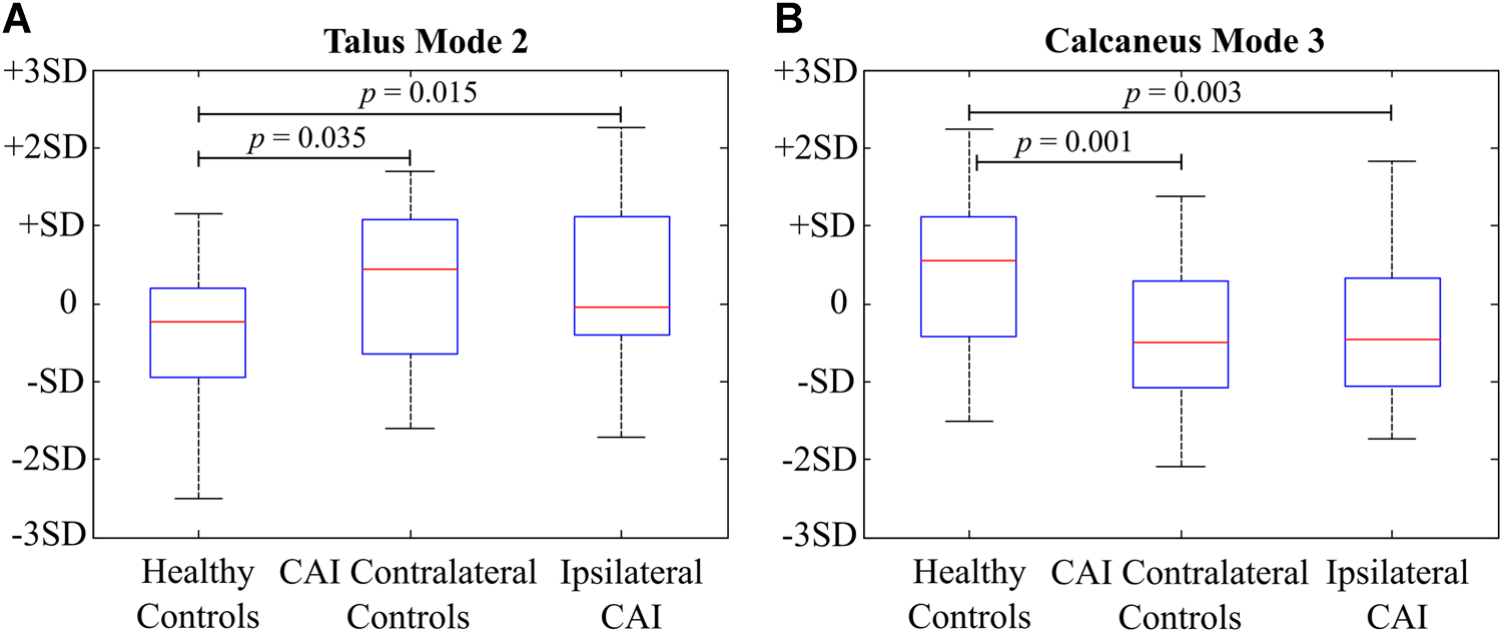
The box plots describing the distributions of the shape parameters for the following groups: ipsilateral CAI, CAI contralateral controls, and healthy controls. These distributions are presented for the Mode 2 of the talus (A) and Mode 3 of the calcaneus (B) The *p*‐values resulting from the Bonferroni post‐hoc test are indicated for the different shape modes. CAI, chronic ankle instability; SD, standard deviation. [Color figure can be viewed at wileyonlinelibrary.com].

**Figure 5:**
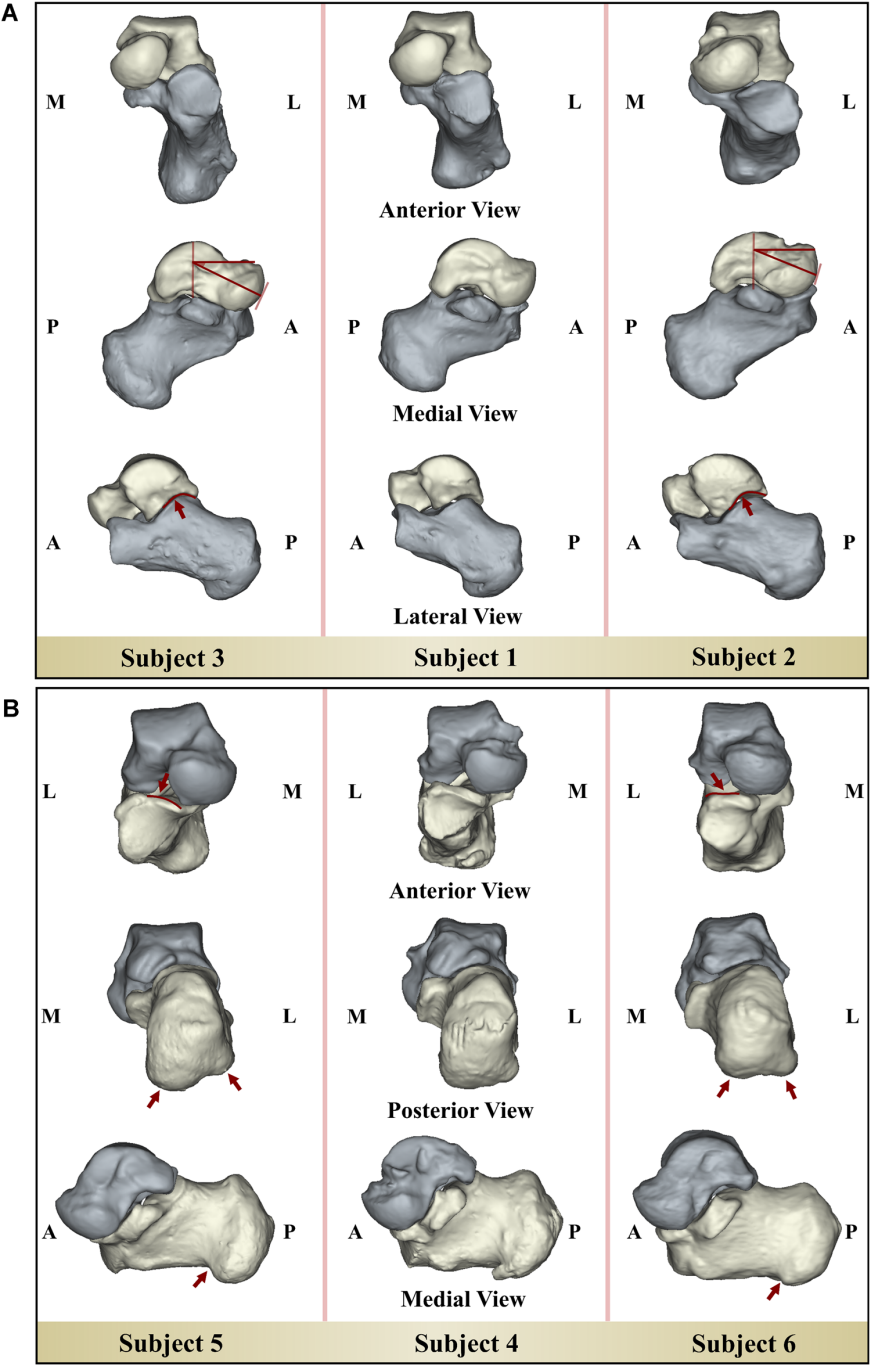
(A) The tali and their calcaneal counterparts of three subjects: Subject 1 whose talus is the closest to the mean talus shape (i.e., shape parameter is close to zero), Subject 2 (i.e., a patient with CAI, shape parameter is +2.3), and Subject 3 (i.e., a healthy control, shape parameter is −2.5) whose tali shapes are the farthest away from the mean talus shape in the positive and negative directions of the shape variation explained by the Mode 2 of the talus, respectively. (B) The calcanei and their tali counterparts of three subjects: Subject 4 whose calcaneus is the closest to the mean calcaneus shape (i.e., shape parameter is close to zero), Subject 5 (i.e., a healthy control, shape parameter is +2.3), and Subject 6 (i.e., a patient with CAI, shape parameter is −2.1) whose calcanei shapes are the farthest away from the mean calcaneus shape in the positive and negative directions of the shape variation explained by the Mode 3 of the calcaneus, respectively. CAI, chronic ankle instability. [Color figure can be viewed at wileyonlinelibrary.com].

The shapes of the tali of the CAI and CAI contralateral groups deviated from the mean talus shape in the positive direction of the Mode 2 of the talus (Fig. 4A). Positive deviations from the mean talus shape describe a decrease in the inclination angle of the talar neck relative to the talar body and a change in the curvature of the talar lateral process that adversely affects the congruency of the articular facets at the posterior side (Fig. 2). Inversely, an increase in the inclination angle of the talar neck relative to the talar body and relatively higher curvature in the talar lateral process were observed in the tali with negative deviations away from the mean talus shape (Fig. 2). Figure 5A shows the tali and their calcaneal counterparts extracted from three subjects.

Shape variations represented by the Mode 3 of the calcaneus (Fig. 3) were significantly different between the following groups: CAI vs. healthy controls (*p* = 0.003) and CAI contralateral controls vs. healthy controls (*p* = 0.001).

While the calcanei of the healthy controls deviated positively from the mean calcaneus shape in Mode 3 of the calcaneus, those of the CAI patients had negative deviations (Fig. 4B). In the CAI patients, the lateral tuberosity stood at the same horizontal line with the medial tuberosity or was relatively more distally positioned (i.e., negative deviation from the mean calcaneus shape, Fig. 3). On the contrary, the healthy subjects had less lateral tuberosity extension (i.e., positive deviation from the mean calcaneus shape, Fig. 3). Additionally, in the calcanei of the CAI patients, the contour of the anterior articular surface was relatively flat as compared with those of the healthy controls (Fig. 3). Fig. 5B shows the calcanei and their talar counterparts derived from three subjects.

Consideration of the age and gender in the statistical analyses as covariates (i.e., ANCOVA) caused no changes in the statistical significance of the reported results.

## DISCUSSION

In this study, a 3D SSM technique was used to compare the shapes of the talus and calcaneus between both sides of the patients with unilateral CAI as well as between CAI patients and healthy controls. The results indicate specific shape differences in the bones. These make individuals with CAI distinguishable from healthy individuals with no known history of ankle joint pathology.

**Figure 6:**
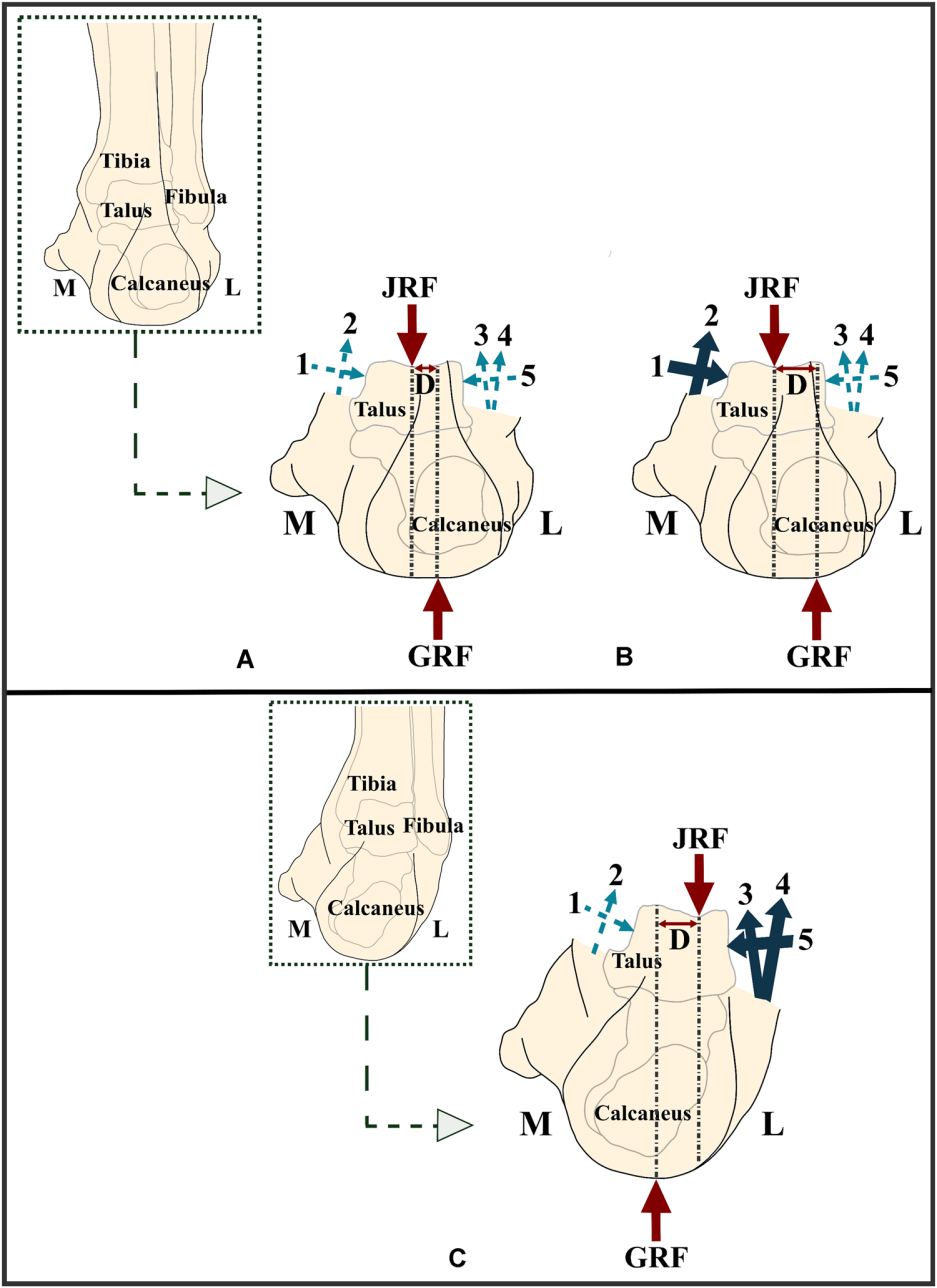
(A) JRF and GRF represent the joint reaction force and ground reaction force, respectively. The light blue dashed and dark blue solid arrows display the forces acting on the subtalar joint (STJ) due to (1) medial malleolar, (2) deltoid ligament, (3) anterofibular ligament, (4) peroneal muscles, and (5) fibula. The dark blue solid arrows represent relatively higher forces as compared with those indicated with the light blue dashed arrows. In a normal foot, there is a distance (*D*) between the axes along which GRF and JRF act. A pair of GRF and JRF mainly generates a moment that effects the STJ. (B) A slight lateral offset of the GRF axis, which can be seen in case the lateral tuberosity of the calcaneus extends more distally, results in an increase in the distance (*D*). In turn, the moment acting on the STJ will increase. (C) With an inversion movement, the effects of the shift of the GRF axis toward the lateral side may be minimized. GRF, ground reaction force; JRF, joint reaction force. [Color figure can be viewed at wileyonlinelibrary.com].

Considering the different types of shape differences detected here, the CAI patients seem to be exhibiting less congruent STJ shapes (i.e., less complex, generally flatter joint surface) as well as a flattened calcaneal ground‐contact surface (Fig. 5B). Variations in the medial and lateral calcaneal tuberosities (Figs. 3 and 5B) can alter the loading moment, which is formed by a pair of the ground reaction force (GRF) and joint reaction force (JRF) (Fig. 6A and B). In healthy subjects, the GRF and JRF axes do not coincide with each other (Fig. 6A) and a pronation exorotation moment occurs.^39^ If the GRF slides laterally (Fig. 6B), which is highly probable if both medial and lateral tuberosities extend to the ground, the distance (*D*) between both axes will increase. In turn, the pronatory external moment will be higher and will cause extra strain on the medial muscles and ligaments. A patient may minimize the lateral shift of the GRF axis and avoid losing balance by an inversion movement (Fig. 6C). This compensating action may lead to a recurrent LAS, if not countered on time.

**Figure 7:**
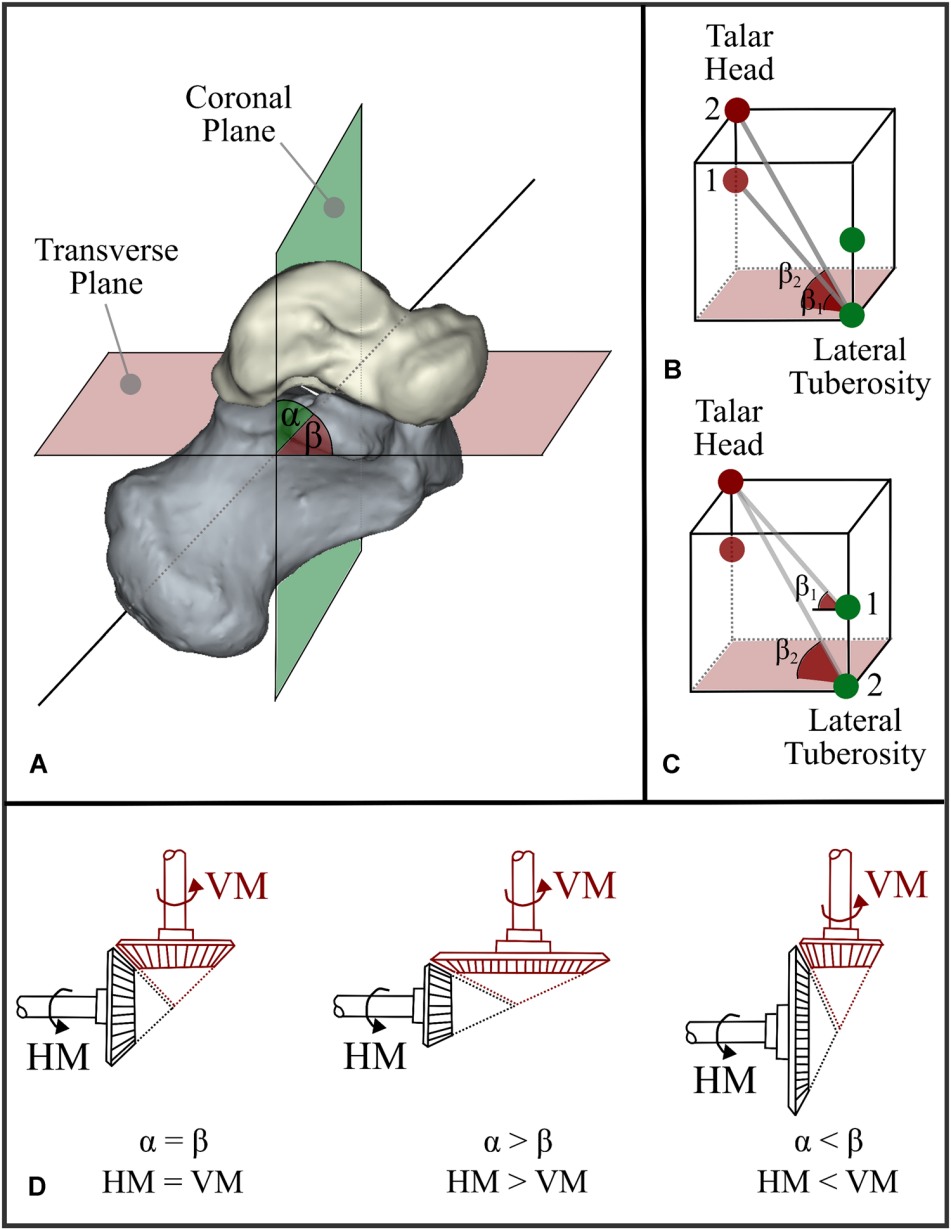
(A) 3D orientation of the STJ axis relative to the hindfoot. *α* and *β* are the angles from the coronal and transverse planes to the STJ axis, respectively. (B) Assuming that the lateral tuberosity of the calcaneus is constant, changes in the talar head/neck (e.g., Point 1 vs. Point 2) can lead to a different orientation of the STJ axis. (C) A similar configuration. This time, it is assumed that the talar head/neck is constant and that the lateral tuberosity of the calcaneus changes. (D) An analogy can be made to a gear mechanism. If the STJ axis passes at 45° (*α* = *β*), the rotations around the vertical axis and the horizontal axis are equal to each other. If the STJ axis is vertically aligned (i.e., *β* > *α*), the rotation around the horizontal axis is less than the one around the vertical axis. In the cases where *β* < *α* (horizontal alignment of the STJ axis), it is the other way around. 3D, three dimensional; HM, horizontal motion; STJ, subtalar joint; VM, vertical motion. [Color figure can be viewed at wileyonlinelibrary.com].

Furthermore, the shape variations found for the talus (Mode 2, Figs. 2 and 5A) and the calcaneus (Mode 3, Figs. 3 and 5B) may affect the 3D orientation of the STJ axis (Fig. 7A). In individuals with CAI, a decrease in the inclination angle of the talar neck relative to the body (Fig. 7B) and/or a distal extension of the lateral tuberosity (Fig. 7C) is more likely, which can cause the STJ to be more vertically oriented (Fig. 7A, *β* > α). Considering that the STJ axis is oblique, an analogy can be made to a gear mechanism shown in Fig. 7D. Similar to the coupling between the vertical and horizontal motions illustrated in these configurations (Fig. 7D), a motion of the STJ in one plane has components in the other two planes. In the case of a more vertically oriented STJ axis (Fig. 7D, *β* > α), less rotation around the horizontal axis of the talus in relation with the calcaneus is observed for a given rotation around the vertical axis (Fig. 7D). The limitation in rotation may increase the risk of losing balance quicker in an inversion motion, as the control of the muscles of the ankle joint ends as soon as the maximal range of motion is exceeded. Several studies have been previously performed to evaluate the relationship between foot deformities and CAI.^8^, ^24^ In individuals with cavus foot deformity,^39^ one of the foot deformities that is known to be associated with CAI, the position of the STJ axis is reported to be more vertically oriented. Shape variations in the talus and calcaneus that increase the tendency for a more vertical axis, as observed for the individuals with CAI, may explain why cavus foot is associated with CAI. Therefore, researchers may be able to clarify some observations and associations that remain unexplained by including bone shape variations in the studies aimed at understanding the relationship between foot deformities and CAI.

Shape variations described by the modes of shape variation beyond 6 for the talus and 5 for the calcaneus were not presented and compared between the groups. Although these modes collectively explain >50% of total shape variation, it is not expected that easily recognizable and important shape variations have been missed. Recognizing and interpreting the shape variations described by the higher modes (i.e., Mode 7 and higher for the talus, and Mode 6 and higher for the calcaneus) is difficult, as they are subtle and are distributed over bone surfaces. Moreover, not all of these modes of shape variation explain meaningful shape variations, as they may simply describe the noise caused by scanning and point sampling.

The main limitation of this study is the retrospective nature of the data collection. Although instability is certainly present in the CAI patients, more concomitant problems (e.g., osteoarthritis) may be the cause of the observed bony shape differences. Moreover, information on the activity level of the patients was not available, which may be a contributing factor as well. The age and gender of the subjects were, however, known. Therefore, the effects of age and gender could be accounted for in our statistical analyses.

Investigating the mechanisms that can lead individuals to develop CAI after sustaining a LAS were outside the scope of the current study. Nevertheless, we attempted to explain some mechanisms that can be associated with shape variations in the STJ bones and may play a role in the onset of CAI. With this perspective, bony shape variations described by Mode 2 of the talus and Mode 3 of the calcaneus were considered. Such explanations are, however, limited in the sense that the SSMs of the talus and calcaneus were developed independently from each other, without having a coupling between them to describe simultaneous shape variations. Irrespective of the exact mechanism, the most important finding of the current study is that two specific talar and calcaneal shape variations appear to be significantly different between CAI patients and healthy controls. Determining whether these findings represent post‐traumatic changes or whether recurrent LAS originate from these variations requires further research in a prospective setting. However, the fact that bone shape did not vary within individuals with one unstable ankle suggests that these shape variations play a role in the development of CAI after a first‐time LAS and are not caused by CAI.

The bone shapes of the subjects with and without CAI overlap. Some of the healthy subjects exhibit geometrical features that are similar to those identified as being typical for the CAI group. These individuals may have not developed CAI yet or may have not sustained an injury that leads to the onset of CAI, but may nevertheless be at the risk of developing CAI. Inversely, some of the CAI patients do not show the shape features that we found to be typical of the CAI group, but had nevertheless sustained events that had led to the genesis of CAI, potentially due to other risk factors.

Within this study, the shape variations in the bones of the TCJ of the CAI patients and healthy controls were not considered. Instead, we focused on the STJ bones and their shape variations. That is due to the fact that the TCJ and its relation with CAI have already been the main focus of researchers, while the STJ and its potential contribution to CAI have been often overlooked. Nevertheless, the methodology presented here is not limited to the STJ bones and could be applied to the TCJ bones as well.

This study is a next step in identifying the risk factors that originate from the shape of our bones. Creating SSMs that are based on a prospective data set would help in assessing the prognostic values of the bone shape variations on the development of CAI. Moreover, if the described shape variations could be translated into reliably measurable parameters for conventional radiology, they can be also included in the risk assessment models that are used in clinical settings. This can provide clinicians with more insight into which treatment options may work the best for individual patients who have sustained LAS.

## CONCLUSION

The 3D statistical shape models of the talus and calcaneus were built based on the mixed data of patients with unilateral CAI and healthy controls. These enabled us to quantitatively compare shape variations between the ipsilateral and contralateral sides of CAI patients, the ipsilateral sides of CAI patients and healthy controls, and between the contralateral sides of CAI patients and healthy controls. We found two specific statistically significant shape differences between CAI patients and healthy controls. In the case of the talus, the identified shape mode affected the curvature of the talar lateral process as well as the inclination angle of the talar neck relative to the body. As for the calcaneus, the identified shape mode was related to the medial and lateral tuberosities combined with the contour of the anterior articular surface.

## AUTHORS’ CONTRIBUTION

All the authors contributed to the study design. G.V. and G.K. contributed materials and G.V. checked the data set to discard CT scans satisfying exclusion criteria. Scans of healthy controls were provided by G.V., G.K., and L.B. L.B. contributed materials and SSM generation tools. N.T. post‐processed the data set and analyzed the data together with G.V., L.B., G.T., and A.Z. All authors revised the paper for intellectual content and approved the final version of the manuscript to be published.

## Supporting information

Supporting information.
