# Supplementary material for: Typical Shape Differences in the Subtalar Joint Bones Between Subjects with Chronic Ankle Instability and Controls
Source: J Orthop Res. 2019 May 26;37(9):1892–902. doi: 10.1002/jor.24336 (PMC6772087; doi:10.1002/jor.24336)
Supplement: Supplementary file 1 — Supporting information. [file JOR-37-1892-s001.docx]

**SUPPLEMENTARY MATERIAL**

**Description of a shape parameter:**

A shape parameter (i.e. b value) for a given mode of variation (i.e. it explains the direction of shape changes) mainly describes how far a shape (**x**) is away from the mean shape ($\bar{\mathbf{x}}$) in the direction of shape changes. For example, the positions of a shape **x** and the mean shape $\bar{\mathbf{x}}$ are shown in blue and red, respectively. The shape parameter b_1_ explains the distance (highlighted in green) between **x** and $\bar{\mathbf{x}}$ in the mode of shape variation $\mathbf{Ф}_{1}$:


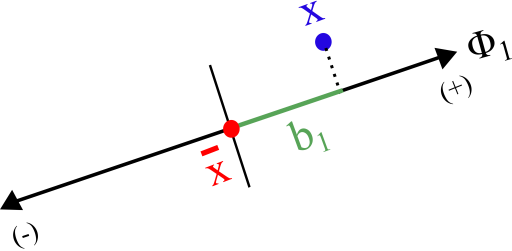


Figure S1. The shape parameter *b*_1_ describes how far the given shape x is away from the mean shape $\bar{\mathbf{x}}$ in the mode of variation $\mathbf{Ф}_{\boldsymbol{1}}$.

**Dense Correspondence Establishment Across Bone Surfaces of the Same Type:**

A dense correspondence between each pair was established using the coordinates of points and surface normals as described in the study^1^. In brief, the correspondence between a point **a**_i_ on surface A and a point **b_ai_** on surface B was defined using the minimum Euclidean distance^1^:

$$\mathbf{b}_{\mathbf{ai}}= {}_{b_{j}}^{\arg min}\left\| \boldsymbol{a}_{i}-\boldsymbol{b}_{j} \right\|$$

where $\boldsymbol{a}_{i}\boldsymbol{=}\left[ \begin{matrix} \mathbf{p}_{i} \\ \mathbf{n}_{i} \end{matrix} \right]$ and $\boldsymbol{b}_{j}\boldsymbol{=}\left[ \begin{matrix} \mathbf{p}_{j} \\ \mathbf{n}_{j} \end{matrix} \right]\boldsymbol{,}\mathbf{p}_{i}$ and $\mathbf{p}_{j}$ represent the point coordinates, while $\mathbf{n}_{i}$ and $\mathbf{n}_{j}$ stand for the surface normals.

**Cumulative Shape Variation in the Talus and Calcaneus:**

Figures S2a and S2b display the cumulative shape variation in the talus and calcaneus with respect to number of modes, respectively.


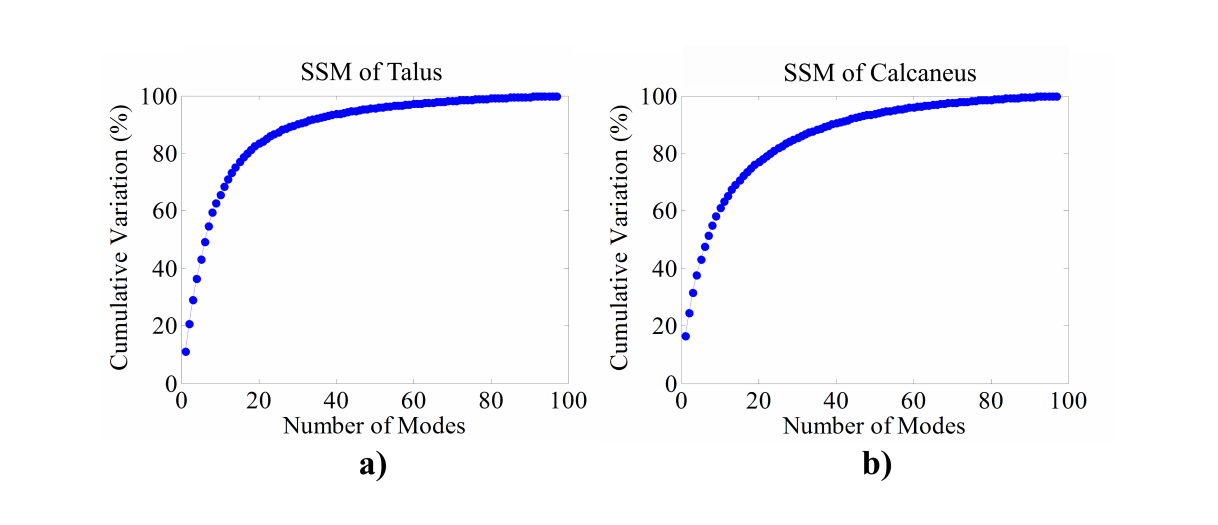


**Figure S2**. Contribution (%) of each shape mode to the total shape variation in the (a) talus and (b) calcaneus.

Following the performance of PCA on the covariance matrix, the two plots shown in Figure S2 were obtained using the eigenvalues and the formula:

$$r= \frac{\sum_{s=1}^{c} \lambda_{s}}{\sum_{s=1}^{N-1} \lambda_{s}} \times100$$

in which, *c* and *N* stand for the number of eigenvalues and the number of samples, respectively. The percentage ratio, *r* , describes the fraction of total variance retained.

**REFERENCES**

1. van de Giessen M, Smitsman N, Strackee SD, et al. A statistical description of the articulating ulna surface for prosthesis design. *Proc - 2009 IEEE Int Symp Biomed Imaging From Nano to Macro, ISBI 2009*. 2009:678-681. doi:10.1109/ISBI.2009.5193138
